# Prevalence and distribution of metabolic syndrome and its components among provinces and ethnic groups in Indonesia

**DOI:** 10.1186/s12889-019-6711-7

**Published:** 2019-04-03

**Authors:** Elizabeth Henny Herningtyas, Tian Sheng Ng

**Affiliations:** 1 Clinical Pathology and Laboratory Medicine Department, Faculty of Medicine, Public Health and Nursing, Universitas Gadjah Mada, Radioputro Building 5th floor, Jalan Farmako, Sekip Utara, Yogyakarta, Indonesia; 2 Undergraduate Program, Faculty of Medicine, Public Health and Nursing, Universitas Gadjah Mada, Grha Wiyata Building, Jalan Farmako, Sekip Utara, Yogyakarta, Indonesia

**Keywords:** Metabolic syndrome prevalence, MetS component distribution, Provinces in Indonesia, Indonesia’s ethnic groups

## Abstract

**Background:**

Global increase of metabolic syndrome (MetS) may have affected Indonesia, however, lack of data in this multiethnic group country warrants a nationwide study for MetS and its components. This study aims to determine the prevalence of metabolic syndrome and its components among Indonesian people based on the province and ethnic groups.

**Methods:**

We obtained 8573 subjects from the Indonesian Family Life Survey Wave 4 (IFLS4), spread over 20 provinces in Indonesia and consisting of 27 ethnic groups. MetS was operationalized according to an adapted Harmonized MetS definition. Prevalence ratios with 95% confidence interval were estimated using log-binomial regression.

**Results:**

The prevalence of MetS in Indonesia is 21.66% with provincial prevalence ranging from 0 to 50%, while the ethnic prevalence ranging from 0 to 45.45%. Significant higher MetS prevalence ratios were found in Jakarta (PR 1.826; 95CI 1.628–2.048), West Nusa Tenggara (PR 1.412; 95CI: 1.222–1.630), West Sumatra (PR 1.404; 95CI: 1.202–1.641), East Java province (PR 1.109; 95CI: 1.001–1.229) and in Sasak (PR 1.532; 95CI:1.304–1.800), Minangkabau (PR 1.469; 95CI:1.251–1.726), Betawi (PR 1.597; 95CI:1.346–1.895), Acehnese ethnic group (PR 2.101; 95CI:1.099–4.020) while significant lower prevalence ratios were observed in Central Java (PR 0.668; 95CI: 0.580–0.770), Yogyakarta (PR 0.695; 95CI: 0.575–0.840), Banten (PR 0.718; 95CI: 0.533–0.968), Bali province (PR 0.724; 95CI: 0.590–0.889) and in Javanese (PR 0.855; 95CI:0.788–0.928), also Balinese ethnic groups (PR 0.669; 95CI:0.535–0.836). The highest prevalence of MetS components among Indonesians was low HDL cholesterol (66.41%), followed by hypertension (64.45%), and central obesity (43.21%).

**Conclusions:**

The prevalence of MetS in Indonesia is moderate with provincial and ethnic prevalence varied. Provincial and ethnic group differences in MetS prevalence ratios were observed. The top two most prevalent MetS components in Indonesian were low HDL cholesterol and hypertension.

**Electronic supplementary material:**

The online version of this article (10.1186/s12889-019-6711-7) contains supplementary material, which is available to authorized users.

## Background

Metabolic syndrome (MetS) is a cluster of interconnected risk factors that increase the risk of cardiovascular disease (CVD) and type 2 diabetes mellitus (T2DM) [1]. Increased prevalence worldwide affects both developed countries as well as developing countries, including the Asia-Pacific region [2]. This MetS increase is in concordance with the 82% increase of the worldwide obesity pandemic over the time span of 1990 to 2010 based on the Global Burden of Disease (GBD) [3]. According to a systemic review in 2007 by Ranasinghe et al. [2], the prevalence of MetS was in the range of 11.9 to 37.1% in the Asia-Pacific region which consists of the Philippines, China, Sri Lanka, Taiwan, Singapore, South Korea, Mongolia and Malaysia with the prevalence of 11.9, 21.3, 24.3, 25.5, 26.9, 31.3, 32.8 and 37.1%, respectively, based on different MetS syndrome definitions.

Among Southeast Asian countries, Indonesia is the most populated country in this region which reached 227 million people in 2005 [4], consisting of various ethnic groups that were estimated by 2007 to be 633 [5], along with various cultures and lifestyles spread over 33 provinces [6]. Since an increasing trend in MetS prevalence was found in other regions, Indonesia might be affected simultaneously, however, little is known regarding the MetS epidemiology in this multiethnic group country.

A few studies have tried to assess the MetS prevalence in certain provinces of Indonesia separately such as in Yogyakarta (13.19%) [7], and Jakarta (28.4%) [8], based on different MetS syndrome definitions but none yet have attempted the assessment in a larger scope such as a national survey. An update for MetS prevalence that represents MetS prevalence in a national scope is warranted and will benefit national planning for future prevention of MetS clinical consequences. Concerning the variations of Indonesian inhabitants and wide distribution of Indonesian lifestyles, it was hypothesized that the distribution of metabolic syndrome prevalence and MetS components may vary among provinces and ethnic groups in Indonesia.

Disability Adjusted Life Years (DALYs) contributed by risk factors associated with MetS including high BMI, high blood pressure, high fasting plasma glucose, high total cholesterol have grown since 1990 to 2000 based on GBD [3]. Low HDL cholesterol is the most prevalent risk factor, followed by central obesity, hypertension, high triglyceride level and high fasting plasma glucose level based on a Korean study in 2011 [9]. The prevalence pattern of MetS risk factors might differ in Indonesia due to different demographic and geographical factors and hence may affect the impact of each component involved in MetS based on different MetS definitions.

Thus, the aim of this study is to determine the prevalence of metabolic syndrome and MetS components among Indonesian people based on the province and ethnic groups.

## Methods

### Study sites

Indonesia is an archipelago country with an estimated population of 266,703,831 in 2018 (226,712,730 in 2005) by United Nations estimates that resides in 1,811,570 km^2^ area and median age of population is 28.3 years [4]. Indonesia consists of 33 provinces in 2007 and 20 out of those 33 provinces were involved in this study [6].

### Data

The participants in IFLS survey gave written informed consent according to the Institutional Review Board in National Institute of Health, USA. The usage of the IFLS datasheet had been approved by Institutional Review Board in Faculty of Medicine, Public Health and Nursing, Universitas Gadjah Mada. We combined data from various sources. For the individual data, we used the Indonesian Family Life Survey (IFLS) 4. The IFLS data were publicly available and can be retrieved in the RAND website (http://www.rand.org/labor/FLS/IFLS.html).

The Indonesian Family Life Survey (IFLS) is a longitudinal survey conducted in Indonesia since 1995 and now reaches the Wave 5 that was conducted in 2013. The Fourth Wave of IFLS consisted of 50,580 subjects who originated from 7224 households by randomized stratified sampling in 1995 and had been expanded to 13,995 by the 2007 survey [10]. The required information in this study included the number of subjects, age, sex, waist circumference (WC), HDL cholesterol, height, weight, body mass index (BMI), systolic and diastolic blood pressure and the usage of medication for hypertension and diabetes. In this survey, the basic information on each household were location, sex, age and socio-demographic characteristics. Additionally, the physical health assessment data, which was done by two specially trained nurses in taking the health related measurements included height and weight in all subjects, waist and hip circumference in subjects 40 years and older, total and HDL cholesterol in subjects 40 years and older, along with blood pressure and pulse in subjects 15 years and older. The cholesterol level was measured by using the CardiochekPA system with capillary blood samples.

The subjects included in this study were aged 40 and above who are Indonesian citizens selected according to the IFLS 4 study, due to lack of WC and HDL-C data for subjects < 40 years old. Subjects with incomplete data for collection of the research variables/risk factors and pregnant were excluded. The data was considered as incomplete data if there was no data on one or more of these variables: provincial code, gender, age, systolic and diastolic blood pressure, HDL-C, WC, ethnic group, and urban/rural status.

We used the Harmonized MetS definition from the Joint Interim Statement [1] which requires three out of five risk factors: central obesity, hypertension, low HDL cholesterol or evidence of cholesterol treatment, elevated triglyceride or evidence of elevated triglyceride treatment, and hyperglycemia or evidence of diabetes treatment. However, due to no data regarding elevated triglycerides, this risk factor was not present in the MetS components list in this study. Hence, the operational definition for this study was adapted by using three out of four existing components. Central obesity was defined by the Asian cut-off that uses the waist circumference < 90 cm for men and < 80 cm for women. Hypertension was defined as systolic blood pressure ≥ 130 mmHg or diastolic BP ≥85 mmHg or treatment of previously diagnosed hypertension. Low HDL-cholesterol was defined as serum HDL-Cholesterol < 40 mg/dL in males, < 50 mg/dL in females or specific treatment for reduced HDL-Cholesterol. Diabetes treatment was employed as a substitute for insulin resistance due to lack of fasting plasma glucose data. Gender is defined as male or female. Age is classified into pre-elderly group (40 - < 65 years old) and elderly group (≥ 60 years old). Ethnicity is classified into Javanese, Sundanese, Balinese, Batak, Buginese, Chinese, Madurese, Sasak, Minangkabau, Banjarese, Bima-Dompu, Makassar, Nias, Palembang, Sumbawa, Toraja, Betawi, Dayak, Malay, Komering, Ambonese, Manado, Acehnese, Sumbagsel, Bantenese, Cirebonese and other ethnic groups. Geographic factor is classified into urban and rural status.

### Statistical analysis

The data were analyzed using Stata v12.0 format software. Continuous variables were expressed as mean ± standard deviation (SD). Student’s t test or Mann-Whitney U-test was used to compare continuous variables. Categorical variables were reported as count and proportions. Proportions were tested using the chi-square test or Fisher’s exact test, whenever appropriate.

Prevalence and prevalence ratio of MetS and their respective 95% confidence intervals (95% CI) were estimated for gender, demographic characteristic, provinces and ethnic groups using log-binomial regression by comparing the reference group with the remaining subjects of the study population.

## Results

### Basic characteristic of study Population

The total amount of individuals in the target household of IFLS 4 was 50,580 subjects, however, only 44,103 subjects were interviewed. Among the 44,103 subjects, only 11,712 subjects age > 40 years old from the IFLS 4 datasheet were included. After excluding subjects with incomplete data and in pregnancy, a total of 8573 subjects were obtained, consisting of 3886 males (45.33%) and 4687 females (54.67%). The baseline characteristics of study population are presented in Table 1. Among the estimated 633 ethnic groups in Indonesia, we obtained 26 major ethnic groups that were spread over 20 provinces out of 33 provinces in Indonesia in year 2007, and classified the remaining minor ethnic group subjects in “Others”.

**Table 1:** Baseline Characteristics of MetS components and demographic components of the study population

Characteristics	Total (%)	MetS (%)	Non-MetS(%)	*p*
Gender (%)				
Male	3886 (45.33)	459 (24.72)	3427 (51.03)	< 0.001
Female	4687 (54.67)	1398 (75.28)	3289 (48.97)	
Age Group				
Pre-elderly	6987 (81.50)	1491 (80.29)	5496 (81.83)	0.129
Elderly	1586 (18.50)	366 (19.71)	1220 (18.17)	
Average age (Mean ± SD)	53.77 ± 10.99	54.48 ± 10.92	53.57 ± 11.01	0.0017
Ethnic Group (%)^a^				
Javanese	3979 (46.41)	790 (42.54)	3189 (47.48)	< 0.001
Sundanese	1022 (11.92)	220 (11.85)	802 (11.94)	
Balinese	461 (5.38)	68 (3.66)	393 (5.85)	
Batak	257 (3)	60 (3.23)	197 (2.93)	
Buginese	303 (3.53)	61 (3.28)	242 (3.60)	
Chinese	87 (1.01)	21 (1.13)	66 (0.98)	
Madurese	285 (3.32)	57 (3.07)	228 (3.39)	
Sasak	329 (3.84)	107 (5.76)	222 (3.31)	
Minangkabau	349 (4.07)	109 (5.87)	240 (3.57)	
Banjarese	215 (2.51)	49 (2.64)	166 (2.47)	
Bima-Dompu	99 (1.15)	21 (1.13)	78 (1.16)	
Makassar	99 (1.15)	28 (1.51)	71 (1.06)	
Nias	34 (0.4)	5 (0.27)	29 (0.43)	
Palembang	32 (0.37)	8 (0.43)	24 (0.36)	
Sumbawa	41 (0.48)	13 (0.70)	28 (0.42)	
Toraja	45 (0.52)	12 (0.65)	33 (0.49)	
Betawi	271 (3.16)	92 (4.95)	179 (2.67)	
Dayak	6 (0.07)	0 (0.00)	6 (0.09)	
Malay	60 (0.7)	12 (0.65)	48 (0.71)	
Komering	6 (0.07)	1 (0.05)	5 (0.07)	
Ambon	9 (0.1)	1 (0.05)	8 (0.12)	
Manado	3 (0.03)	1 (0.05)	2 (0.03)	
Acehnese	11 (0.13)	5 (0.27)	6 (0.09)	
Sumbagsel#	274 (3.2)	53 (2.85)	221 (3.29)	
Bantenese	26 (0.3)	8 (0.43)	18 (0.27)	
Cirebonese	161 (1.88)	26 (1.40)	135 (2.01)	
Others	109 (1.27)	29 (1.56)	80 (1.19)	
Geographic factor (%)				
Urban	4526 (52.79)	1140 (61.39)	3386 (50.42)	< 0.001
Rural	4047 (47.21)	717 (38.61)	3330 (49.58)	
Anthropometric Parameter (Mean ± SD)				
Height (m)	1.540 ± 0.08353	1.527 ± 0.08056	1.544 ± 0.08396	< 0.0001
Weight (kg)	56.15 ± 10.86	62.51 ± 11.89	54.40 ± 9.863	< 0.0001
BMI (kg/m^2^)	23.62 ± 3.952	26.71 ± 4.001	22.77 ± 3.487	< 0.0001
WC (cm)	82.83 ± 10.79	92.63 ± 8.509	80.12 ± 9.741	< 0.0001
Blood Pressure (Mean ± SD)				
Systolic BP (mmHg)	140.1 ± 24.32	154.4 ± 22.58	136.2 ± 23.28	< 0.0001
Diastolic BP (mmHg)	83.38 ± 11.98	90.32 ± 11.37	81.46 ± 11.43	< 0.0001
Other Characteristics (Mean ± SD or %)				
HDL-Cholesterol (mg/dl)	40.56 ± 14.43	39.50 ± 9.084	42.23 ± 15.18	< 0.0001
Cholesterol treatment	19 (0.22)	13 (0.70)	6 (0.09)	< 0.001
Diabetes treatment	70 (0.82)	59 (3.18)	11 (0.16)	< 0.001
Hypertension treatment	216 (2.52)	110 (5.92)	106 (1.58)	< 0.001

^a^Ethnic group names are based on Demography of Indonesia’s Ethnicity book and IFLS 4 codebook’s original name; #Sumbagsel in southern Sumatra consists of Jambi and Bengkulu ethnic groups

There were significant differences in demographic factors such as gender, average age, ethnic groups, and geographic factor between metabolic syndrome’s group and non-metabolic syndrome group as well as anthropometric and laboratory characteristics. Metabolic syndrome group showed significant heavier body weight (62.51 ± 11.89 vs. 54.40 ± 9.863), larger BMI (26.71 ± 4.001 vs. 22.77 ± 3.487), larger waist circumference (92.63 ± 8.509 vs. 80.12 ± 9.741), higher systolic pressure (154.4 ± 22.58 vs. 136.2 ± 23.28) and diastolic blood pressure (90.32 ± 11.37 vs. 81.46 ± 11.43), lower HDL cholesterol level (39.50 ± 9.084 vs. 42.23 ± 15.18), higher proportion of subjects obtained lipid-lowering medication (0.7% vs. 0.09%), diabetes treatment (3.18% vs. 0.16%) and hypertension treatment (5.92% vs. 1.58%) than non-metabolic syndrome group (Table 1).

### Mapping of metabolic syndrome prevalence across Indonesia

**Fig. 1:**
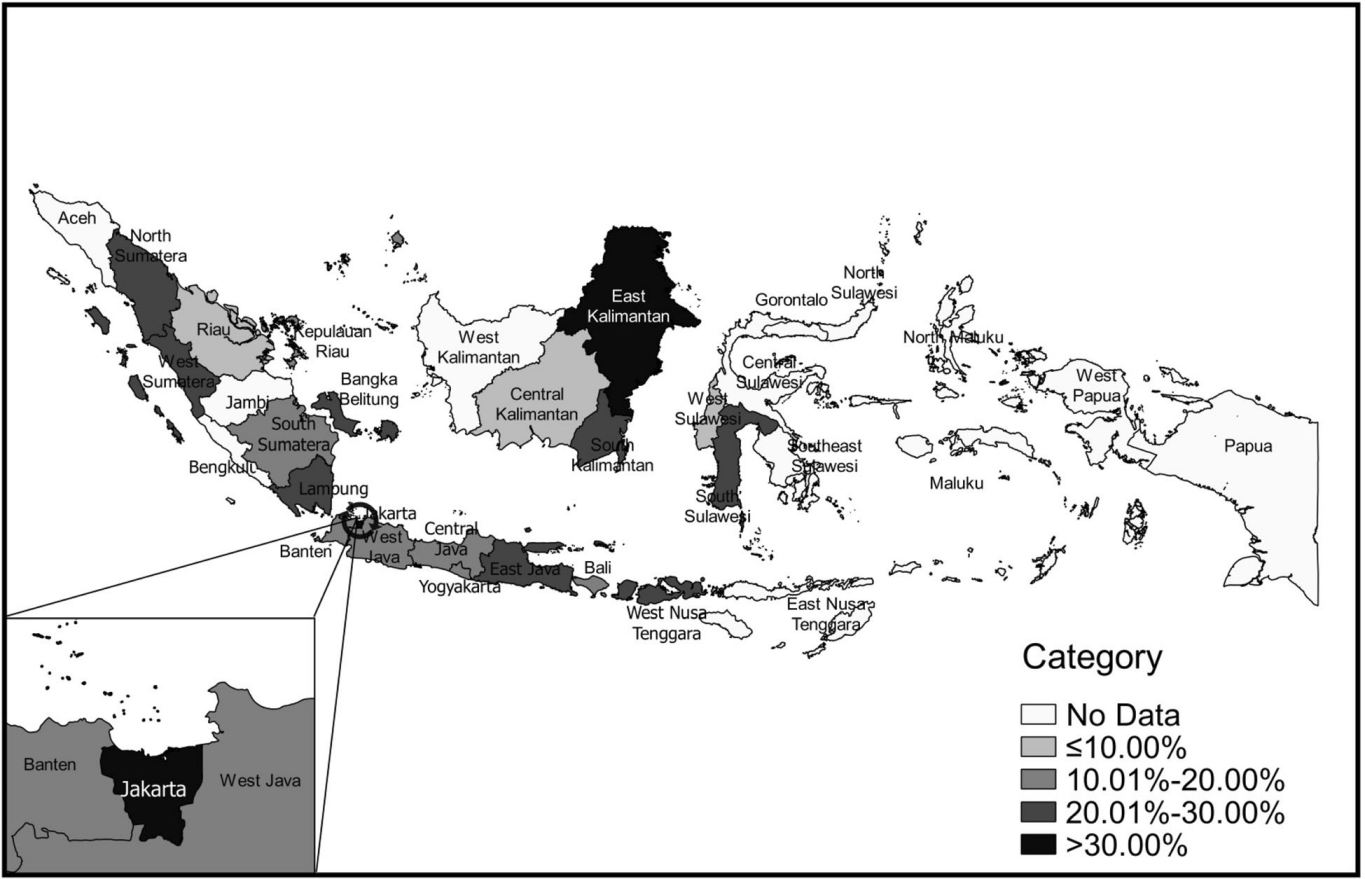
Categorical Distribution of Metabolic Syndrome Prevalence among Provinces in Indonesia. Indonesia Map 2007 was obtained from www.big.go.id by request, further processed using the QGIS v2.18.10. Permission was obtained from Lutfan Lazuardi, MD. PhD for publication of the map

Among the studied provinces, most of the provinces (85%) were located in the west part of Indonesia with higher population count which is comprised of 7 provinces on Sumatra island, 6 provinces on Java island, the Bali province and 3 provinces on Kalimantan island; while the east part of Indonesia was represented by 3 provinces that are comprised of 2 provinces on Sulawesi island and the small archipelago of West Nusa Tenggara province (Fig. 1).

When categorized in percentile categories, the map showed that 2 provinces in Indonesia had MetS prevalence higher than 30% which were Jakarta, and East Kalimantan; 8 provinces had MetS prevalence in the range of 20.01–30% (North Sumatra, West Sumatra, Lampung, Bangka Belitung, East Java, West Nusa Tenggara, South Kalimantan, and South Sulawesi), while 7 provinces had MetS prevalence in the range of 10.01–20% (South Sumatra, Kepulauan Riau, West Java, Central Java, Yogyakarta, Banten, and Bali), and 3 provinces had MetS prevalence less than 10% (Riau, West Sulawesi, and Central Kalimantan) (Fig. 1).

### Distribution of ethic groups among provinces in Indonesia

**Fig. 2:**
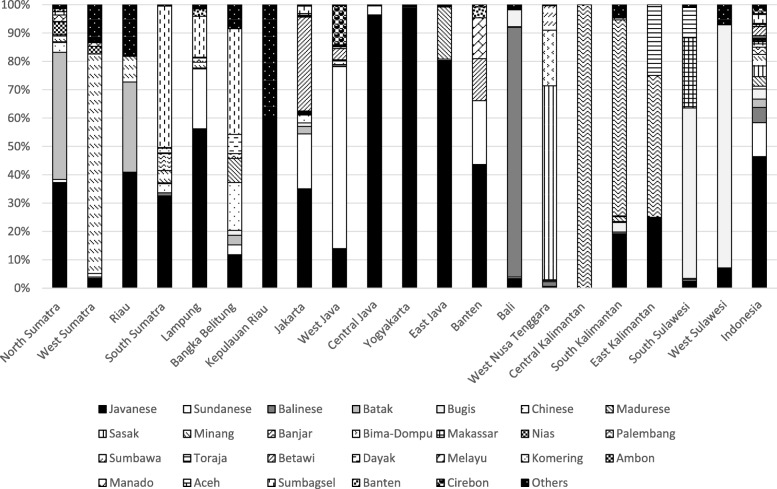
Distribution of ethnic groups among provinces in Indonesia

From the estimated 633 ethnic groups in Indonesia, we identified 27 ethnic groups that were spread over and inhabited 20 provinces. Among those identified ethnic groups, the Javanese ethnic group was the dominant one that reached approximately 46% of Indonesian inhabitants and lived in 19 out of 20 provinces with the highest percentage (99%) in the Special Region of Yogyakarta. The Sundanese was the second highest ethnic group in Indonesia inhabiting 12 out of 20 provinces but most of them live in West Java (Fig. 2).

The other ethnic groups were distributed over 20 provinces with certain ethnic groups tending to be major inhabitants in certain provinces such as the Batak ethnic group was mostly residing in North Sumatra, Minang ethnic group in West Sumatra, Balinese in the Bali province, Bugis in South Sulawesi, and Sasak ethnic group in West Nusa Tenggara (Fig. 2).

### Prevalence and prevalence ratio of metabolic syndrome among provinces and ethnic groups

The prevalence of MetS in Indonesia was 21.66% (Table 2). The MetS prevalence was shown to be more than or equal to 20% in 11 out of 20 provinces in Indonesia (Table 2) and in 19 out of 27 ethnic groups (Table 3). The majority of the subjects are found on Java Island (Banten, Jakarta, West Java, Central Java, Yogyakarta and East Java), covering more than 50% of the total subjects, with MetS prevalence ranging from 15.16 to 37.50%. followed by Sumatra Island (North Sumatra, West Sumatra, Riau, South Sumatra, Lampung, Bangka Belitung, and Kepulauan Riau) ranging from 9.09 to 29.85%, Lesser Sunda Islands (Bali, West Nusa Tenggara) ranging from 15.94 to 29.89%, Sulawesi Island (South Sulawesi and West Sulawesi) ranging from 7.14 to 22.83%, and lastly Kalimantan Island (Central Kalimantan, South Kalimantan, and East Kalimantan) ranging from 0 to 50% (Table 2).

**Table 2:** Metabolic Syndrome Prevalence and Prevalence Ratio among Provinces in Indonesia

Province Code	Province	Number of Subjects	MetS Prevalence, % (95% CI)	MetS Prevalence Ratio, % (95% CI)
12	North Sumatra	488	20.70 (17.09–24.30)	0.953 (0.797–1.139)
13	West Sumatra	402	29.85 (25.36–34.34)	1.404 (1.202–1.641)^a^
14	Riau	22	9.09 (− 3.96–22.14)	0.419 (0.112–1.572)
16	South Sumatra	369	19.78 (15.70–23.87)	0.910 (0.738–1.122)
18	Lampung	340	20.59 (16.27–24.91)	0.949 (0.767–1.173)
19	Bangka Belitung	59	22.03 (11.14–32.93)	1.017 (0.628–1.647)
21	Kepulauan Riau	5	20.00 (−35.53–75.53)	0.923 (0.160–5.332)
31	Jakarta	568	37.50 (33.51–41.49)	1.826 (1.628–2.048)^a^
32	West Java	1141	19.54 (17.24–21.85)	0.889 (0.784–1.008)
33	Central Java	1174	15.16 (13.11–17.21)	0.668 (0.580–0.770)^a^
34	Yogyakarta	617	15.40 (12.54–18.25)	0.695 (0.575–0.840)^a^
35	East Java	1454	23.59 (21.41–25.77)	1.109 (1.001–1.229)^a^
36	Banten	236	15.68 (11.01–20.35)	0.718 (0.533–0.968)^a^
51	Bali	502	15.94 (12.72–19.15)	0.724 (0.590–0.889)^a^
52	West Nusa Tenggara	475	29.89 (25.76–34.03	1.412 (1.222–1.630)^a^
62	Central Kalimantan	2	0	0
63	South Kalimantan	298	23.83 (18.96–28.69)	1.104 (0.897–1.358)
64	East Kalimantan	4	50.00 (−41.87–141.87)	2.310 (0.866–6.159)
73	South Sulawesi	403	22.83 (18.71–26.94)	1.057 (0.879–1.270)
76	West Sulawesi	14	7.14 (−8.29–22.57)	0.329 (0.050–2.180)
	**Indonesia**	**8573**	**21.66 (20.79–22.55)**	

Reference group: total population – targeted group

^a^Statistically significant prevalence ratio

The boldface in this table is emphasizing the total prevalence of MetS in Indonesia

**Table 3:** Metabolic syndrome prevalence and prevalence ratio based on demographic and ethnic group in Indonesia

Characteristic		Subjects Number	MetS Prevalence, % (95% CI)	MetS Prevalence Ratio, % (95% CI)
Gender	Male	3886 (45.33)	11.81 (10.80–12.83)	0.396 (0.360–0.436)^a^
	Female	4687 (54.67)	29.83 (28.52–31.14)	2.525 (2.293–2.781)^a^
Age group	Pre-elderly	6987 (81.50)	21.05 (20.38–22.30)	0.925 (0.836–1.022)
	Elderly	1586 (18.50)	23.29 (21.00–25.15)	1.081 (0.978–1.196)
Geographic factor	Urban	4526 (52.79)	25.19 (23.92–26.45)	1.422 (1.308–1.545)^a^
	Rural	4047 (47.21)	17.72 (16.54–18.89)	0.703 (0.647–0.764)^a^
Ethnic Group	Javanese	3979 (46.41)	19.85 (18.61–21.09)	0.855 (0.788–0.928)^a^
	Sundanese	1022 (11.92)	21.53 (19.00–24.05)	0.993 (0.877–1.245)
	Balinese	461 (5.38)	14.75 (11.50–18.00)	0.669 (0.535–0.836)^a^
	Batak	257 (3)	23.35 (18.14–28.55)	1.080 (0.862–1.353)
	Buginese	303 (3.53)	20.13 (15.59–24.67)	0.927 (0.738–1.164)
	Chinese	87 (1.01)	24.14 (14.96–33.31)	1.116 (0.767–1.623)
	Madurese	285 (3.32)	20.00 (15.33–24.67)	0.921 (0.727–1.166)
	Sasak	329 (3.84)	32.52 (27.43–37.61)	1.532 (1.304–1.800)^a^
	Minangkabau	349 (4.07)	31.23 (26.35–36.12)	1.469 (1.251–1.726)^a^
	Banjarese	215 (2.51)	22.79 (17.14–28.44)	1.054 (0.821–1.352)
	Bima-Dompu	99 (1.15)	21.21 (13.02–29.41)	0.979 (0.668–1.434)
	Makassar	99 (1.15)	28.28 (19.26–37.31)	1.310 (0.955–1.798)
	Nias	34 (0.4)	14.71 (2.16–27.25)	0.678 (0.301–1.525)
	Palembang	32 (0.37)	25.00 (9.13–40.86)	1.155 (0.633–2.107)
	Sumbawa	41 (0.48)	31.71 (16.84–46.58)	1.467 (0.934–2.303)
	Toraja	45 (0.52)	26.67 (13.23–40.10)	1.233 (0.758–2.004)
	Betawi	271 (3.16)	33.95 (28.27–39.62)	1.597 (1.346–1.895)^a^
	Dayak	6 (0.07)	0	0
	Malay	60 (0.7)	20.00 (9.58–30.42)	0.923 (0.555–1.533)
	Komering	6 (0.07)	16.67 (−26.18–59.51)	0.769 (0.128–4.606)
	Ambon	9 (0.1)	11.11 (−14.51–36.73)	0.513 (0.081–3.255)
	Manado	3 (0.03)	33.33 (− 110.09–176.76)	1.539 (0.310–7.630)
	Acehnese	11 (0.13)	45.45 (10.37–80.54)	2.101 (1.099–4.020)^a^
	Sumbagsel	274 (3.2)	19.34 (14.64–24.05)	0.890 (0.696–1.137)
	Bantenese	26 (0.3)	30.77 (11.76–49.78)	1.422 (0.798–2.535)
	Cirebonese	161 (1.88)	16.15 (10.40–21.89)	0.742 (0.521–1.057)
	Others	109 (1.27)	26.61 (18.18–35.03)	1.232 (0.900–1.687)

Reference group: total population – targeted group

^a^Statistically significant prevalence ratio.

The prevalence ratio among provinces showed that 4 provinces had significant increment on the prevalence ratio which were Jakarta (PR 1.826; 95CI: 1.628–2.048), West Nusa Tenggara (PR 1.412; 95CI: 1.222–1.630), West Sumatra (PR 1.404; 95CI: 1.202–1.641), and East Java (PR 1.109; 95CI: 1.001–1.229), respectively, while another 4 provinces had significant decrement on the prevalence ratio which were Central Java (PR 0.668; 95CI: 0.580–0.770), Yogyakarta (PR 0.695; 95CI: 0.575–0.840), Banten (PR 0.718; 95CI: 0.533–0.968) and Bali (PR 0.724; 95CI: 0.590–0.889), respectively (Table 2).

Based on the demographic factor, the prevalence ratio of demographic factors showed a significant higher prevalence ratio in females (PR 2.525; 95CI: 2.293–2.781), and in urban areas (PR 1.422; 95CI: 1.308–1.545) but no difference of prevalence ratio between pre-elderly and elderly. While based on ethnic groups, the significant higher prevalence ratios were found in Sasak (PR 1.532; 95CI: 1.304–1.800), Minangkabau (PR 1.469; 95CI: 1.251–1.726), Betawi (PR 1.597; 95CI: 1.346–1.895), and Acehnese ethnic group (PR 2.101; 95CI: 1.099–4.020). On the contrary, a significant lower prevalence ratio was observed in Javanese (PR 0.855; 95CI: 0.788–0.928), and the Balinese ethnic group (PR 0.669; 95CI: 0.535–0.836) (Table 3).

### Distribution of metabolic syndrome components among provinces and ethnic groups in Indonesia

**Fig. 3:**
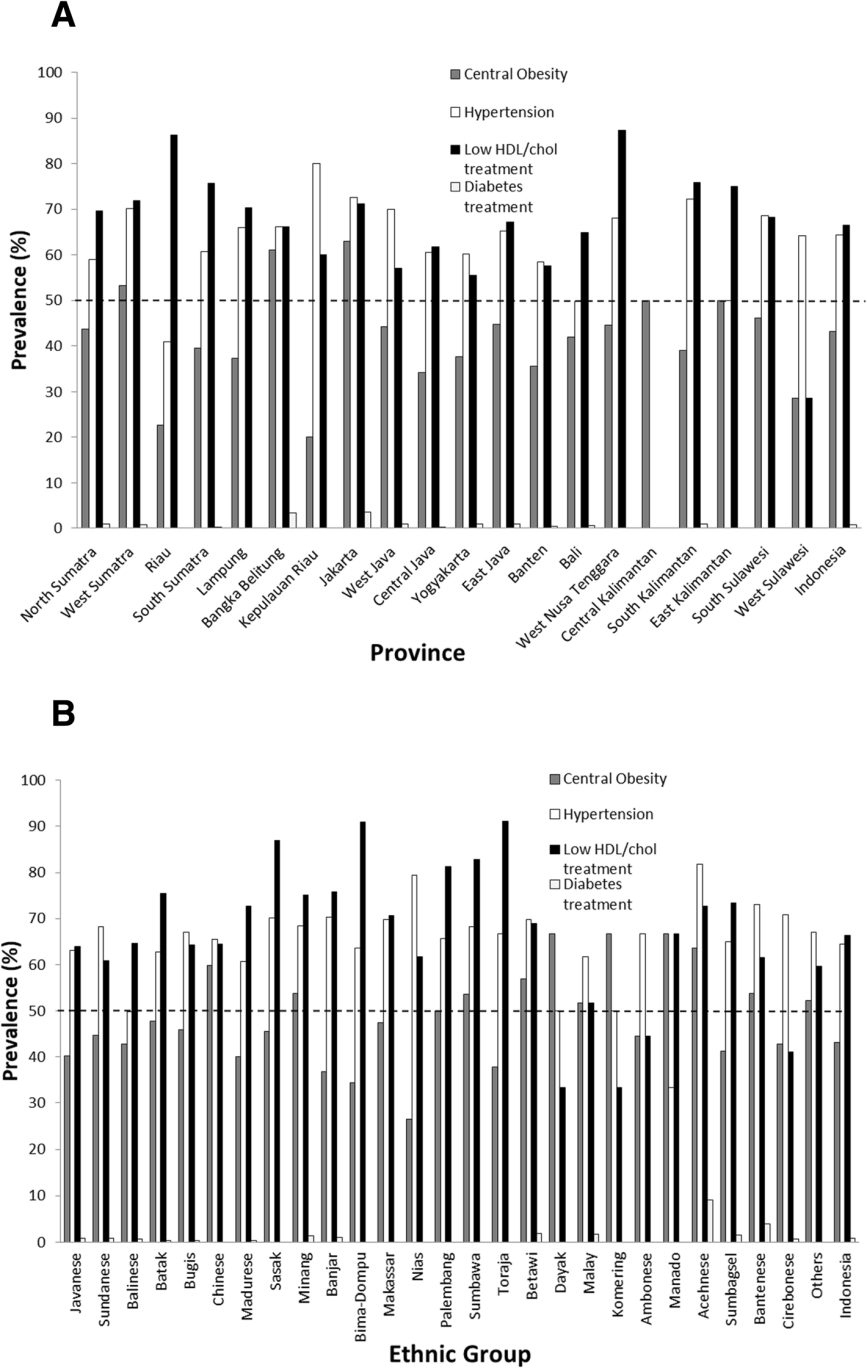
Distribution of Metabolic Syndrome Components based on Province (**a**) and Ethnic Group (**b**)

The highest prevalence of MetS component in Indonesia was low HDL cholesterol (66.41%), followed by hypertension (64.45%), central obesity (43.21%) and insulin resistance (0.82%) (Fig. 3).

Among the observed MetS components in this study, the most prevalent of MetS component was low HDL cholesterol which was found at prevalence of more than 50% in 18 out of 20 provinces in Indonesia and in 23 out of 27 ethnic groups involved (Fig. 3), while hypertension prevalence was found to be more than 50% in 16 out of 20 provinces in Indonesia and in 23 out of 27 ethnic groups (Fig. 3). Central obesity prevalence was found to be more or equal to 50% in 5 out of 20 provinces in Indonesia and in 12 out of 27 ethnic groups (Fig. 3). Diabetes treatment prevalence did not exceed 10% in any provinces in Indonesia nor in any ethnic groups (Fig. 3).

We had tried to determine MetS using 1, 2, 3, or 4 out of four components as the operational definition and we found that using one component made 91.78% of total subjects (7868 out of 8573 subjects) classified as MetS subjects, two components defined 61.11% of the total subjects (5239 out of 8573 subjects) as the MetS group, three components determined 21.66% of the total subjects as MetS subjects, and four components only defined 28 subjects as MetS subjects. (Additional file 1).

The two components definition gave around 39.45% prevalence differences and almost 3 fold prevalence increment compared to three components definition. When we used the two components definition for demographic factors, provinces and ethnic groups, we found similar increment pattern (Additional files 2, 3 and 4). Thus, these findings potentially overestimated the MetS prevalence if we used two components definition and might greatly deviate from the ‘true’ MetS prevalence in Indonesia. Therefore, our current three components definition was more realistic although underestimation possibility was present. Hence, three out of four risk factors remained to be the operational definition in this study.

## Discussion

This research is the first nationwide study regarding MetS prevalence in Indonesia. The prevalence of MetS in Indonesia was 21.66%, which was in concordance with the estimation of global MetS prevalence (20–25%) by IDF [11]. In the Asia-Pacific region, the highest prevalence was found to be in Malaysia (42.5%, Harmonized definition) [12], followed by Mongolia (32.8%, IDF) [13], South Korea (31.3%, Harmonized definition) [14], China (29.7, NCEP ATP III) [15], Singapore (26.9, Harmonized Definition) [16], Taiwan (25.5, Modified NCEP ATP III) [17], Sri Lanka (24.3, IDF) [18], and Philippine (18.6%, Modified NCEP ATP III) [19], so MetS prevalence in Indonesia is the second lowest in Asia Pacific region based on this study.

There is a significant gap between MetS prevalence in Western countries such as USA (39.0, IDF) [20] compared to MetS prevalence in Indonesia (21.66%). It was shown that the national MetS prevalence in Indonesia (21.66%) is less than the urban prevalence of MetS in Jakarta (28.4%) [8], being the capital of Indonesia, hence the latter could not represent the overall prevalence of MetS in Indonesia as demographic factors play significant roles in MetS prevalence. Nevertheless, the prevalence of MetS in Jakarta in this study was 37.5% based on the 2007 survey, which showed higher prevalence compared to the previous study in Jakarta (28.4%) [8], based on the 2006 survey. Therefore, this prevalence difference gave an impression that there is a rising trend in MetS prevalence in Indonesia throughout the years.

Large MetS prevalence difference was seen in Kalimantan Island which was partly due to the limited amount of subjects representing each province. Some subjects were excluded in this study since their data were incomplete, which further reduced the sample size pool. The highest prevalence of MetS when compared among provinces was found in East Kalimantan (50%) followed by Jakarta (37.50%). Despite having only a small number of subjects in East Kalimantan who represented the province, the results were accepted because the data obtained from IFLS 4 are based on stratified random sampling which aimed to ensure estimates can be done with equal accuracy since population disparities were evident across provinces in Indonesia. This trend was consistent with one study which shown that Asians are more likely to manifest MetS compared to non-Hispanic Whites [21] especially when Indonesians had gradually adopted western lifestyles including diet.

As most subjects in this study were residing on Java island, it was not surprising that Javanese was the largest ethnic group in this study which represented 46.41% of the total subjects. Each province tend to have its own unique ethnic group which was the dominant population. This dominant residency pattern might contribute to disparity in MetS prevalence among ethnic groups since each ethnic group might have different lipid profiles, socioeconomics and lifestyle, similar as in the previous studies [22–24]. The prevalence of MetS in Balinese is 0.67 times less than the remaining population, followed by Javanese which was 0.86 times less than the remaining population, showing lesser prevalence in these two ethnic groups. The prevalence of MetS in Acehnese was 2.1 times greater than the remaining population, followed by Sasak (1.53 times greater), Betawi (1.60 times greater) and Minangkabau (1.469 times greater). This finding tallied with the results from a study regarding lipid profiles among four ethnic groups including Minangkabau, Sundanese, Javanese and Buginese, revealed the Minangkabau ethnic group has the highest total plasma cholesterol and lowest plasma LDL cholesterol, Sundanese have the lowest plasma HDL cholesterol and Javanese have the lowest total and LDL plasma cholesterol and highest HDL plasma cholesterol [22]. Since the Javanese group had the best lipid profile, it is consonant with the finding in this study which shown MetS prevalence is significantly lower in the Javanese ethnic group compared to others. In a related study lipid profiles were consistently associated with insulin resistance because insulin affects the metabolism of HDL plasma cholesterol and triglycerides [25], hence central obesity is further developed with poor glucose and lipid control.

The top 3 most prevalent MetS components in this study were low HDL cholesterol, followed by hypertension and central obesity. This pattern was similar to one of the South America countries, Brazil, with low HDL cholesterol as the most prevalent component (59.3%), followed by hypertension (52.5%), and central obesity (38.9%) [26]. However, European countries such as Poland, displayed a different pattern starting from top to bottom, abdominal obesity (75.1%), hypertension (71%) and insulin resistance (37.3%) [27]. In Asian countries such as China, the most prevalent component was hypertension (24.52%), followed by dyslipidemia (24%) and central obesity (22.07%) [28]. Furthermore, Malaysia showed a reverse arrangement for the top 3 components, which were abdominal obesity (57.4%), hypertension (52.3%) and low HDL cholesterol (42.7%) [12]. Each country held a different pattern for the prevalence of each metabolic syndrome component, and even Malaysia as a neighbor country in the same continent showed dissimilarity, hence it is unlikely to have a standard pattern framed on each population due to wide variations of cultural background, demographic and socioeconomic factors.

Even when the low HDL cholesterol, hypertension and central obesity’s pattern was brought forward to compare among Indonesia provinces, this pattern did not apply in certain provinces such as Kepulauan Riau, Jakarta, West Java, Yogyakarta, Banten, Central Kalimantan, South Sulawesi and West Sulawesi (8 out of 20 province). It was also different in certain ethnic groups such as Sundanese, Bugis, Chinese, Nias, Toraja, Dayak, Malay, Komering, Ambonese, Manado, Acehnese, Bantenese, Cirebonese and other minority ethnic groups (14 out of 27 ethnic groups). This pattern disparity definitely had effect on the criteria used to define MetS. Central obesity was not the most prevalence component, hence by using the IDF criteria which required central obesity as a core component [11] might potentially leave out MetS subjects, which reduces the sensitivity of screening the population. However, the specific racial cut-off values for waist circumference is valuable for Asian populations as NCEP ATP III criteria had a higher cut-off value for which the latter might not include the potential MetS subjects. Therefore, the Harmonized definition was applied in this study which required 3 out of 5 risk factors in addition to specific racial cut-off values for waist circumference. This approach was consistent with the finding that shown the Harmonized definition had the highest sensitivity [29] compared to IDF and NCEP ATP III criteria. The Body Mass Index (BMI) for defining obesity in Asian populations is also lower than in Caucasian populations since the percentage of body fat is higher in Asians, as Asian population are more likely to develop T2DM and CVD at BMI ranging 22-25 kg/m^2^ [30], therefore it is necessary to implement lower cut-off values for waist circumference in Asian populations.

Limitations of this study include the lack of plasma triglyceride data which reduced one component from the 5 risk factors. Low HDL plasma cholesterol level does not indicate the presence of high plasma triglyceride in Indonesians, which is consistent with the finding based on a study which shown low HDL plasma cholesterol and normal triglyceride level in most of the West Africans and American Africans manifested with MetS [31]. Hence, it is possible to find a pattern of high plasma triglyceride level with normal HDL plasma cholesterol subjects who may not be classified as MetS subjects. There is also no fasting plasma glucose data which reduces the hyperglycemia sample size pool, rendering the possibility of leaving out potential MetS subjects with hyperglycemia. For the cholesterol treatment, the drug class is not specified in the datasheet. Thus, we cannot differentiate whether the treatment was meant for reduced HDL-cholesterol, elevated triglyceride or elevated LDL-cholesterol. Limited amount of subjects in certain provinces further reduces the specificity of MetS prevalence, which probably results in an over-estimate or under-estimate of the MetS prevalence in those provinces. Since this study showed approximately 1 MetS Indonesian were found among every 5 Indonesians in year 2007, this finding was alarming that the exact national MetS prevalence might be higher than estimated. Furthermore, the potential underestimation of this study was definitely more superior than extrapolating from sporadic provincial studies which were deemed inconclusive.

Since this is the first nationwide study of MetS prevalence, population based strategies should be implemented to control the expansion of MetS prevalence. MetS subjects had higher medical care utilization and costs compared to non-MetS subjects which increase approximately 24% in costs with each additional risk factor [32]. Furthermore, the stakeholders related to IFLS could include fasting plasma glucose and plasma triglyceride levels in future data collections because both components are important health determinants related to CVD and T2DM and more accurate MetS incidence can be determined.

## Conclusion

The prevalence of MetS in Indonesia is moderate with provincial and ethnic prevalence varied. Provincial and ethnic group differences in MetS prevalence ratios were observed. The top two most prevalent MetSomponents in Indonesian were low HDL cholesterol and hypertension.

## Additional files

Additional file 1: Determination of MetS prevalence by the presence of Components Number. (DOCX 12 kb)

Additional file 2: Metabolic syndrome prevalence and prevalence ratio based on demographic and ethnic group in Indonesia when using 2 component as MetS definition. (DOCX 16 kb)

Additional file 3: Distribution of Metabolic Syndrome Components based on Province. (DOCX 16 kb)

Additional file 4: Distribution of Metabolic Syndrome Components based on Ethnic Group. (DOCX 15 kb)

## Abbreviations

BMI: Body Mass Index
CVD: Cardiovascular Disease
DALYs: Disability Adjusted Life Years
GBD: Global Burden of Disease
HDL: High Density Lipoprotein
IDF: International Diabetes Federation
IFLS: Indonesian Family Life Survey
LDL: Low Density Lipoprotein
MetS: Metabolic Syndrome
NCEP ATP III: National Cholesterol Education Program Adult Treatment Panel III
T2DM: Type 2 Diabetes Mellitus
WC: Waist Circumference
