# Supplementary material for: Prevalence and distribution of metabolic syndrome and its components among provinces and ethnic groups in Indonesia
Source: BMC Public Health. 2019 Apr 3;19:377. doi: 10.1186/s12889-019-6711-7 (PMC6448251; doi:10.1186/s12889-019-6711-7)
Supplement: Supplementary file 1 — Determination of MetS prevalence by the presence of Components Number. (DOCX 12 kb) [file 12889_2019_6711_MOESM1_ESM.docx]

Additional File 1. Number of Components and MetS Prevalence (%)

| **Number of Components** | **n (%)** |
| --- | --- |
| **1** | 7868 (91.78) |
| **2** | 5239 (61.11) |
| **3** | 1857 (21.66) |
| **4** | 28 (0.33) |
