# Supplementary material for: Prevalence and distribution of metabolic syndrome and its components among provinces and ethnic groups in Indonesia
Source: BMC Public Health. 2019 Apr 3;19:377. doi: 10.1186/s12889-019-6711-7 (PMC6448251; doi:10.1186/s12889-019-6711-7)
Supplement: Supplementary file 2 — Metabolic syndrome prevalence and prevalence ratio based on demographic and ethnic group in Indonesia when using 2 component as MetS definition. (DOCX 16 kb) [file 12889_2019_6711_MOESM2_ESM.docx]

Additional File 2. Metabolic syndrome prevalence and prevalence ratio based on demographic and ethnic group in Indonesia when using 2 component as MetS definition

| Characteristic | | Subjects Number | MetS Prevalence, % | MetS Prevalence Ratio.  % (95% CI) |
| --- | --- | --- | --- | --- |
| Gender | Male  Female | 3886 (45.33)  4687 (54.67) | 49.92  70.39 | 0.709 (0.684 - 0.736)*  1.410 (1.359 - 1.462)* |
| Age group | Pre-elderly  Elderly | 6987 (81.50)  1586 (18.50) | 59.88  66.52 | 0.900 (0.865 - 0.937)*  1.111 (0.587 - 0.610)* |
| Geographic factor | Urban  Rural | 4526 (52.79)  4047 (47.21) | 63.65  58.27 | 1.902 (1.056 - 1.130)*  0.915 (0.885 - 0.947)* |
| Ethnic Group | Javanese | 3,979 (46.41) | 57.75 | 0.902 (0.872 - 0.934)* |
|  | Sundanese | 1,022 (11.92) | 61.15 | 1.000 (0.950 - 1.054) |
|  | Balinese | 461 (5.38) | 53.15 | 0.863 (0.791 - 0.942)* |
|  | Batak | 257 (3) | 67.32 | 1.105 (1.013 - 1.205)* |
|  | Buginese | 303 (3.53) | 64.69 | 1.061 (0.974 - 1.155) |
|  | Chinese | 87 (1.01) | 71.26 | 1.168 (1.021 - 1.336)* |
|  | Madurese | 285 (3.32) | 60.70 | 0.993 (0.903 - 1.092) |
|  | Sasak | 329 (3.84) | 74.16 | 1.224 (1.146 - 1.308)* |
|  | Minangkabau | 349 (4.07) | 72.78 | 1.201 (1.123 - 1.283)* |
|  | Banjarese | 215 (2.51) | 65.58 | 1.075 (0.974 - 1.186) |
|  | Bima-Dompu | 99 (1.15) | 68.69 | 1.126 (0.984 - 1.287) |
|  | Makassar | 99 (1.15) | 64.65 | 1.059 (0.914 - 1.226) |
|  | Nias | 34 (0.4) | 55.88 | 0.914 (0.678 - 1.233) |
|  | Palembang | 32 (0.37) | 75.00 | 1.228 (1.005 - 1.501)* |
|  | Sumbawa | 41 (0.48) | 75.61 | 1.239 (1.040 - 1.475)* |
|  | Toraja | 45 (0.52) | 71.11 | 1.164 (0.966 - 1.404) |
|  | Betawi | 271 (3.16) | 68.63 | 1.128 (1.039 - 1.224)* |
|  | Dayak | 6 (0.07) | 50.00 | 0.818 (0.367 -1.821) |
|  | Malay | 60 (0.7) | 56.67 | 0.927 (0.742 - 1.157) |
|  | Komering | 6 (0.07) | 66.67 | 1.091 (0.619 - 1.922) |
|  | Ambon | 9 (0.1) | 55.56 | 0.909 (0.507 - 1.631) |
|  | Manado | 3 (0.03) | 66.67 | 1.091 (0.490 - 2.429) |
|  | Acehnese | 11 (0.13) | 81.82 | 1.339 (1.013 - 1.771)* |
|  | Sumbagsel | 274 (3.2) | 64.23 | 1.053 (0.962 - 1.152) |
|  | Bantenese | 26 (0.3) | 69.23 | 1.133 (0.877 - 1.465) |
|  | Cirebon | 161 (1.88) | 52.80 | 0.862 (0.744 - 0.998)* |
|  | Others | 109 (1.27) | 62.39 | 1.021 (0.882 - 1.183) |

Reference group: total population – targeted group

*Statistically significant prevalence ratio
