# Supplementary material for: Prevalence and distribution of metabolic syndrome and its components among provinces and ethnic groups in Indonesia
Source: BMC Public Health. 2019 Apr 3;19:377. doi: 10.1186/s12889-019-6711-7 (PMC6448251; doi:10.1186/s12889-019-6711-7)
Supplement: Supplementary file 3 — Distribution of Metabolic Syndrome Components based on Province. (DOCX 16 kb) [file 12889_2019_6711_MOESM3_ESM.docx]

Additional File 3. Distribution of Metabolic Syndrome Components based on Province

| **MetS Components**  **Province** | | **Central Obesity (%)** | **Hypertension (%)** | **Low HDL chol or chol treatment (%)** | **Diabetes treatment (%)** | **Total number of subjects (n)** |
| --- | --- | --- | --- | --- | --- | --- |
| 12 | North Sumatra | 43.65 | 59.02 | 69.67 | 1.02 | 488 |
| 13 | West Sumatra | 53.23 | 70.15 | 71.89 | 0.75 | 402 |
| 14 | Riau | 22.73 | 40.91 | 86.36 | 0 | 22 |
| 16 | South Sumatra | 39.57 | 60.7 | 75.61 | 0.27 | 369 |
| 18 | Lampung | 37.35 | 65.88 | 70.29 | 0 | 340 |
| 19 | Bangka Belitung | 61.02 | 66.1 | 66.1 | 3.39 | 59 |
| 21 | Kepulauan Riau | 20 | 80 | 60 | 0 | 5 |
| 31 | Jakarta | 63.03 | 72.54 | 71.13 | 3.52 | 568 |
| 32 | West Java | 44.26 | 69.94 | 57.06 | 0.88 | 1141 |
| 33 | Central Java | 34.07 | 60.56 | 61.75 | 0.26 | 1174 |
| 34 | Yogyakarta | 37.6 | 60.29 | 55.59 | 0.97 | 617 |
| 35 | East Java | 44.77 | 65.27 | 67.19 | 0.89 | 1454 |
| 36 | Banten | 35.59 | 58.47 | 57.63 | 0.42 | 236 |
| 51 | Bali | 42.03 | 49.8 | 64.94 | 0.6 | 502 |
| 52 | West Nusa Tenggara | 44.63 | 68 | 87.37 | 0 | 475 |
| 62 | Central Kalimantan | 50 | 0 | 0 | 0 | 2 |
| 63 | South Kalimantan | 38.93 | 72.15 | 75.84 | 1.01 | 298 |
| 64 | East Kalimantan | 50 | 50 | 75 | 0 | 4 |
| 73 | South Sulawesi | 46.15 | 68.49 | 68.24 | 0 | 403 |
| 76 | West Sulawesi | 28.57 | 64.29 | 28.57 | 0 | 14 |
| INA | Indonesia | 43.21 | 64.45 | 66.41 | 0.82 | 8573 |
