# Supplementary material for: Prevalence and distribution of metabolic syndrome and its components among provinces and ethnic groups in Indonesia
Source: BMC Public Health. 2019 Apr 3;19:377. doi: 10.1186/s12889-019-6711-7 (PMC6448251; doi:10.1186/s12889-019-6711-7)
Supplement: Supplementary file 4 — Distribution of Metabolic Syndrome Components based on Ethnic Group. (DOCX 15 kb) [file 12889_2019_6711_MOESM4_ESM.docx]

Additonal File 4. Distribution of Metabolic Syndrome Components based on Ethnic Group

|  | **Ethnic group** | **Central Obesity (%)** | **Hypertension (%)** | **Low HDL chol or chol treatment (%)** | **Diabetes treatment (%)** | **Total number of subjects (n)** |
| --- | --- | --- | --- | --- | --- | --- |
| 1 | Javanese | 40.26 | 63.16 | 63.96 | 0.9 | 3,979 |
| 2 | Sundanese | 44.62 | 68.2 | 60.76 | 0.78 | 1,022 |
| 3 | Balinese | 42.73 | 49.89 | 64.64 | 0.65 | 461 |
| 4 | Batak | 47.86 | 62.65 | 75.49 | 0.39 | 257 |
| 5 | Bugis | 45.87 | 67 | 64.36 | 0.33 | 303 |
| 6 | Chinese | 59.77 | 65.52 | 64.37 | 0 | 87 |
| 7 | Maduranese | 40 | 60.7 | 72.63 | 0.35 | 285 |
| 8 | Sasak | 45.59 | 70.21 | 86.93 | 0 | 329 |
| 9 | Minang | 53.87 | 68.48 | 75.07 | 1.43 | 349 |
| 10 | Banjar | 36.74 | 70.23 | 75.81 | 0.93 | 215 |
| 11 | Bima-Dompu | 34.34 | 63.64 | 90.91 | 0 | 99 |
| 12 | Makassar | 47.47 | 69.7 | 70.71 | 0 | 99 |
| 13 | Nias | 26.47 | 79.41 | 61.76 | 0 | 34 |
| 14 | Palembang | 50 | 65.63 | 81.25 | 0 | 32 |
| 15 | Sumbawa | 53.66 | 68.29 | 82.93 | 0 | 41 |
| 16 | Toraja | 37.78 | 66.67 | 91.11 | 0 | 45 |
| 17 | Betawi | 56.83 | 69.74 | 69 | 1.85 | 271 |
| 18 | Dayak | 66.67 | 50 | 33.33 | 0 | 6 |
| 19 | Melayu | 51.67 | 61.67 | 51.67 | 1.67 | 60 |
| 20 | Komering | 66.67 | 50 | 33.33 | 0 | 6 |
| 21 | Ambon | 44.44 | 66.67 | 44.44 | 0 | 9 |
| 22 | Manado | 66.67 | 33.33 | 66.67 | 0 | 3 |
| 23 | Aceh | 63.64 | 81.82 | 72.73 | 9.09 | 11 |
| 25 | Sumbagsel | 41.24 | 64.96 | 73.36 | 1.46 | 274 |
| 26 | Banten | 53.85 | 73.08 | 61.54 | 3.85 | 26 |
| 27 | Cirebon | 42.86 | 70.81 | 40.99 | 0.62 | 161 |
| 95 | Others | 52.29 | 66.97 | 59.63 | 0 | 109 |
| INA | Indonesia | 43.21 | 64.45 | 66.41 | 0.82 | 8573 |
